# Situational Awareness: The Effect of Stimulus Type and Hearing Protection on Sound Localization

**DOI:** 10.3390/s21217044

**Published:** 2021-10-24

**Authors:** Leah Fostick, Nir Fink

**Affiliations:** Department of Communication Disorders, Ariel University, Ariel 40700, Israel; nirfi@ariel.ac.il

**Keywords:** localization, speech, mirror image reversal errors

## Abstract

The purpose of the current study was to test sound localization of a spoken word, rarely studied in the context of localization, compared to pink noise and a gunshot, while taking into account the source position and the effect of different hearing protection devices (HPDs) used by the listener. Ninety participants were divided into three groups using different HPDs. Participants were tested twice, under with- and no-HPD conditions, and were requested to localize the different stimuli that were delivered from one of eight speakers evenly distributed around them (starting from 22.5°). Localization of the word stimulus was more difficult than that of the other stimuli. HPD usage resulted in a larger mean root-mean-square error (RMSE) and increased mirror image reversal errors for all stimuli. In addition, HPD usage increased the mean RMSE and mirror image reversal errors for stimuli delivered from the front and back, more than for stimuli delivered from the left and right. HPDs affect localization, both due to attenuation and to limitation of pinnae cues when using earmuffs. Difficulty localizing the spoken word should be considered when assessing auditory functionality and should be further investigated to include HPDs with different attenuation spectra and levels, and to further types of speech stimuli.

## 1. Introduction

The ability to localize sound is important for survival, originating from the need to identify potential risks in the environment, whether a stalking predator or an approaching car when crossing a street. Sound localization is derived mostly from a comparison of information perceived by both ears, such as differences in the precise time at which the sound reaches each ear (interaural time difference, ITD), and the intensity of the sound reaching each ear (interaural level difference, ILD) [1]. While ITD is more effective for localizing low frequencies (lower than 1500 Hz), and ILD is more effective for localizing high frequencies (higher than 1500 Hz), frequencies in the range of 2000–4000 Hz are poorly localized [2,3]. In addition to differences between ears, information is also obtained about the location of a sound from spectral changes that occur when it encounters body parts such as the torso, head, and pinnae [1,4]. The accuracy in localizing sound depends on the characteristics of the environment, the listener, and the stimulus.

In environmental characteristics, one basic feature among many is the spatial angle from which an acoustic stimulus is delivered relative to the listener’s forehead or relative to another point of reference. In this study, we focused on acoustic stimuli presented in the horizontal plane, thus referring to horizontal positions (left, right, front, back, or any other azimuth) in reference to the forehead of a listener who is seated in the center and surrounded by equidistant sound sources. Studies have shown that the localization of stimuli delivered from the front, left, or right (relative to the forehead direction) is more accurate than those delivered from the back [5,6,7]. Confusing a right-delivered stimulus for a left one, or vice versa (termed here as right/left or left/right errors), reflects a difficulty in perceiving cues related to interaural differences. Confusing a front-delivered stimulus for a back one, or vice versa (termed here as front/back or back/front errors), reflects a difficulty in perceiving spectral cues, related to changes in the sound’s spectra due to the pinnae, head, and torso [5,6]. Spectral cues contribute less than interaural difference cues to localization [1,8], but the localization of sound sources from the front or the back is especially difficult when some spectral cues are unavailable, such as when the pinnae are covered by earphones [4,5,6,7,8,9]. Thus, when considering the ability to localize sound, one should consider the source’s azimuth in regard to the listener’s forehead direction. The localization can be evaluated either by specifying the sound source azimuth in units of degrees, or by a binary generalization of the sound source hemifield. The latter reflects whether a stimulus originating from one of the hemifields would be perceived correctly or incorrectly as originating from the opposite hemifield.

The listeners’ hearing ability is another factor affecting sound localization. The contribution of hearing ability to localize sound is usually evaluated among hearing-impaired listeners e.g., [10,11,12] or among typically hearing listeners using hearing protection devices (HPDs) e.g., [4,5,6,7,8,13,14,15]. The present study was of the latter type. HPDs affect the listener’s ability to localize sound in two fundamental ways. First, they *attenuate* sound intensity, causing the sound to be less clear and distinctive, thus having a detrimental effect on the localization of the sound source [4,5,6,7,8,13,14,15]. This underscores the importance of considering the advantages and disadvantages of using HPDs with different attenuation levels [16]. Second, as mentioned earlier, some HPDs (e.g., earmuffs) cover the pinnae, thereby *reducing spectral cues* important for localizing sources emanating from in front of or behind the listener. Earmuffs therefore increase front/back or back/front confusion in localization [4,6,8]. Accordingly, when considering the effect of HPDs on localization, it is important to consider not only their attenuation, but also their type—whether in-the-ear (insert earplugs), over-the-ear (earmuffs), or a combination (double hearing protection), as the latter attributes to a larger attenuation and reduction in spectral cues. Therefore, in the present study, we compared the effects of in-the-ear, over-the-ear, and double-protection HPDs on sound localization.

Lastly, localization also depends on sound characteristics: level (amplitude), duration, and frequency (spectrum). The sound level affects localization ability by causing the sound to be more or less clear and distinctive, as discussed earlier in the context of HPDs. The sound duration affects localization accuracy [8,17] as a longer duration provides greater temporal information and energy than sounds of a shorter duration. In addition, long-duration sounds (more than ~1.5 s) provide the listener with sufficient time to move the head in the direction of the sound, thereby improving localization, particularly by reducing front/back or back/front confusion [1,8,18]. The sound spectrum affects localization accuracy via the frequency range (low-, high-, or middle-range frequencies) and bandwidth (narrow- vs. broad-band spectrum). Low frequencies are localized more accurately than high frequencies [3,19] but high frequencies increase the localization accuracy of sounds presented from the back of the listener [20,21]. In general, broad-band sounds are easier to localize than narrow-band sounds are (assuming equal energy in both), and pure tones are the most difficult to localize [3,7,22,23,24,25].

In everyday life settings, the ability to identify the location of a person addressing us is critical. In such settings, the sound stimulus to be localized will most likely be either a single word (short-duration stimulus) or a sentence (long-duration stimulus). Yet, the localization of words has rarely been studied [23,24,25], and has mainly been explored in the context of hearing aids and cochlear implants [11,12]. In contrast, pink noise e.g., [8,11,26], pure tones, and other narrow- or broad-band noise stimuli [3,4,6,8,10,15,17,25,27,28,29,30] have been repeatedly tested in localization studies, and studies relating to military and sporting activities (such as hunting) specifically focused on the localization of gunshots [7,31,32,33,34]. The few studies that tested the ability to localize different speech stimuli showed that speech stimuli characterized by a narrower bandwidth than pink noise or white noise were poorly localized in comparison, yet better localized than pure tones [11,23,24,25]. However, these few studies did not examine the effect of the environment on word localization, such as the azimuth of the sound relative to the listener, nor the listener’s hearing ability.

Therefore, the aim of the current study was to test the ability to localize a monosyllabic word stimulus compared to two other stimuli with different spectra that have been frequently studied in previous localization studies, namely, pink noise and a gunshot sound. The localization of these stimuli was also tested in relation to the azimuth of the sound source and with the participants using various HPDs, thus manipulating hearing ability and spectral cues.

## 2. Materials and Methods

### 2.1. Participants

A total of 90 individuals participated in the study, aged 20–35 years (58% females). The participants were undergraduate students, recruited using advertisements within campus social networks, and screened for normal hearing (hearing thresholds ≤25 dB HL in frequencies of 500, 1000, 2000, 3000, 4000, 6000, and 8000 Hz). Exclusion criteria also included a diagnosis of a learning disability or attention deficit hyperactivity disorder. Participants were randomly divided into three groups of 30 participants each, in order to measure the effect of the three HPDs.

### 2.2. Stimuli

**Figure 1:**
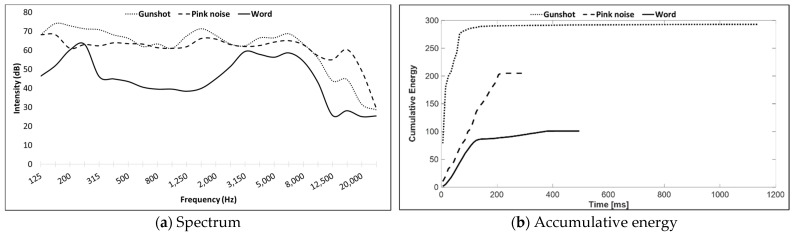
Description of Hebrew word /esh/ (fire), pink noise, and M16 single gunshot at a distance of 200 feet (60.96 m).

Three stimuli were compared in the current study: the Hebrew word ‘esh’ (fire) spoken by a male speaker, pink noise, and a single M16 assault rifle shot recorded at a distance of 200 feet (60.96 m) from the shooter. The word was recorded in a sound-treated booth, using an Electro-Voice^™^ RE320 microphone connected to a Focusrite^™^ Saffire Pro^™^ 24 DSP sound card, with a Hewlett Packard^®^ computer running MAGIX^®^ Samplitude^®^ Pro X software. The M16 assault rifle shot was recorded by Sintonizar Productions [35]. Pink noise was generated using the Sound Forge™ Pro version 11 software of MAGIX^®^. The duration of the three stimuli was 409 ms for the word, 212 ms for the pink noise, and 202 ms for the gunshot with an additional reverberation tail of 800 ms. For determining the spectrum of each stimulus in the listener’s ear, a GRAS™ 45CB Acoustic Test Fixture (ATF) was placed in the same setting as the head of the listener in the experiment. Stimuli administered from the speaker at an azimuth of 0° were recorded and analyzed using SINUS™ SAMURAI™ version 3.0.2^®^ software. The data are presented in Figure 1a, with the word having the narrowest spectrum and pink noise the widest. Figure 1b presents the accumulative energy for each stimulus, with the word having the least accumulated energy over time, and the gunshot the most.

### 2.3. Hearing Protection Devices

**Figure 2:**
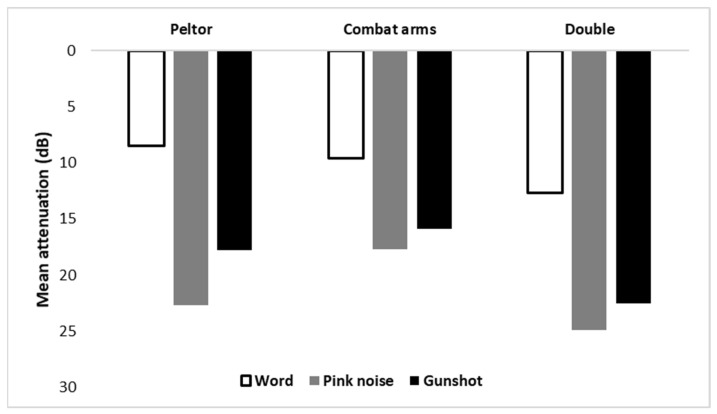
Mean attenuation of word, pink noise, and gunshot by HPD condition (Combat Arms^™^ earplugs, Peltor^™^ earmuffs, and double protection).

Three HPD conditions were tested in the current study. The HPDs were both manufactured by 3M^™^: Combat Arms^™^ 4.1 earplugs in an open mode were used for the in-the-ear condition, 3M^™^ Peltor^™^ Bull’s Eye^™^ H515FB flat earmuffs were used for over-the-ear condition, and the combination was used for the double-protection condition. According to the manufacturer, the Noise Reduction Rating (NRR) is 7 dB for the Combat Arms^™^ earplugs in open mode and is 27 dB for the Peltor^™^ H515FB earmuffs. Figure 2 shows the mean attenuation of each stimulus according to the various HPD configurations used in the study, as recorded by the ATF. The mean attenuation for each stimulus and HPD was calculated by subtracting the mean SPL measured for the unoccluded condition from the mean SPL measured for the occluded condition. As expected, double protection added an attenuation of at least 3 dB to single protection conditions.

### 2.4. Apparatus

**Figure 3:**
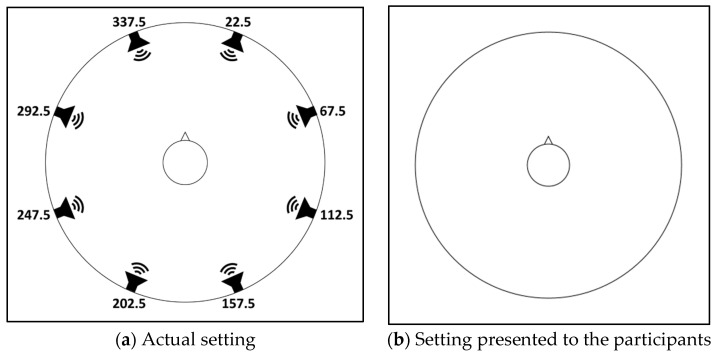
Experimental setting. The symbol in the middle represents participant orientation. (**a**) The actual setting: Participants sat in the middle of a circle of eight monitors separated by 45°, starting from 22.5° through 337.5°. (**b**) The setting presented to participants on a computer screen: participants were asked to indicate on the circle the location of the perceived sound source.

The experiment was carried out using a Dell^™^ Inspiron^™^ 13 5378 i5 laptop computer with designated software that controlled sound delivery and recorded participant responses. The software was written in C# version NET 4.5.2. The sounds were delivered from the computer through a Steinberg^™^ UR824 USB 2.0 Audio Interface into eight RCF Ayra 5^®^ active monitors, positioned 60 cm from participants and separated by 45° starting from 22.5° through 337.5° (Figure 3a). The experimental setup was calibrated by injecting each monitor separately with a 1 kHz tone and measuring 100 dB SPL with the GRAS^™^ 45CB ATF, which was designed and specified to comply with the ANSI/ASA S12.42 standard. The ATF was seated in the center of the monitors’ circle, resembling the position of a human participant in this experiment. The calibration tone signal from each monitor was recorded with two 1/2’’ microphones situated at the end of the ATF’s simulated ear canals (the position of the eardrum in a human) that were connected to a SINUS^™^ SAMURAI^™^ sound level meter conforming to IEC 60651/IEC 60804/IEC 61672-1, IEC 651, and IEC 804 standards.

### 2.5. Procedure

The study was carried out according to Good Clinical Practice (GCP) regulations and was approved by the university institutional review board. The experiment was conducted in a sound-proof anechoic chamber. The participants sat with the computer in a tablet position on their lap. The tablet’s screen presented a top-of-the-head with a tip-of-the-nose symbol representing the participant and their orientation. The participant symbol was surrounded by a circle of continuous circumference that did not indicate the monitor positions (Figure 3b). Participants were asked to respond to each stimulus by indicating the perceived location of each sound source on the circle’s circumference. Each stimulus was delivered 10 times from each monitor, resulting in 240 trials (3 stimuli type × 8 monitors × 10 repetitions) randomly intermixed by the experimental software. After every 48 trials, the participants were offered a short break.

Following the provision of signed informed consent and completing the screening procedure, the participants had a short training session in which all sounds were randomly delivered once from each monitor. A break was offered after training and prior to the beginning of the experiment. Half of the participants were tested first with the HPD condition and then with the no-HPD condition; the other half was tested in the opposite order. The HPDs were fitted by the experimenter. The live experiment duration lasted approximately 20 min for both conditions, with the entire procedure (including screening and training) lasting almost 60 min. Upon completion of the task, participants received monetary compensation equivalent to USD 65 for their time.

### 2.6. Data Analysis

**Figure 4:**
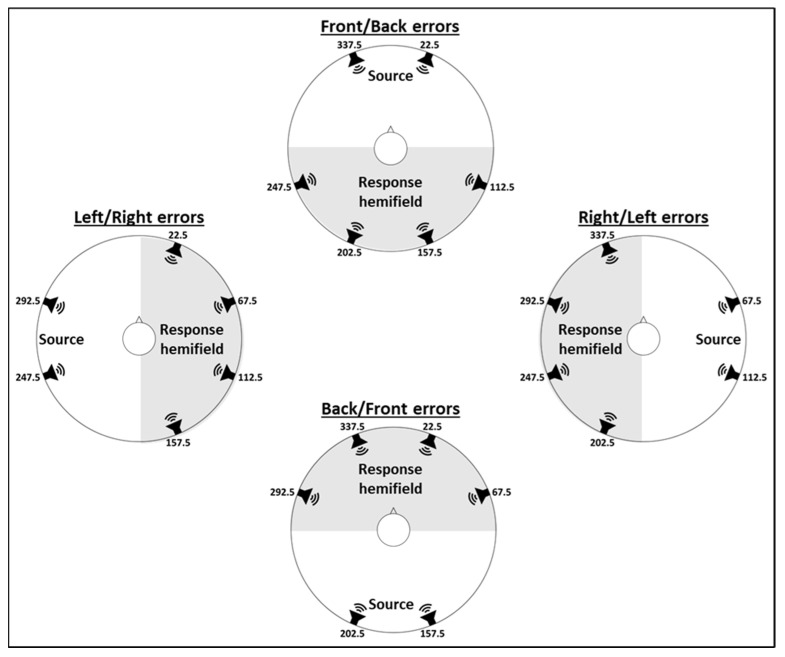
Mirror image reversal errors for each of the four hemifields. Errors were defined by a response opposite the hemifield of the source monitor, as indicated by the shaded areas in each hemifield.

Localization accuracy was analyzed in two ways. First, we analyzed the *discriminating accuracy* of the specific perceived location of each sound source. This was analyzed in terms of the root-mean-square error (RMSE), i.e., the RMSE of the angular distance between the response angle and the target monitor angle. Second, we analyzed *mirror image reversal errors* (see also [9]); this analysis showed the degree to which participants had difficulty discriminating between sound sources due to mirror image reversal errors. Thus, the analysis focused on the percent of errors participants made when they localized the stimuli to the hemifield opposite the target monitors. Therefore, when the target monitors were on the right side (i.e., 67.5° and 112.5°), Right/Left (R/L) mirror image reversal errors were defined as responses of angles 181° to 359°, and when the target monitors were on the left side (i.e., 247.5° and 292.5°), Left/Right (L/R) mirror image reversal errors were defined as responses of angles 1° to 179°. When target monitors were in the front (i.e., 337.5° and 22.5°), Front/Back (F/B) mirror image reversal errors were defined as responses of angles 91° to 269°, and when target monitors were in the back (i.e., 157.5° and 202.5°), Back/Front (B/F) mirror image reversal errors were defined as responses of angles 271° to 360° and 0° to 89° (Figure 4).

Repeated measures ANOVAs were performed with stimuli type (word, pink noise, and gunshot) and hemifield (front, back, right, and left for hemifield discrimination) or source angle (22.5°, 67.5°, 112.5°, 157.5°, 202.5°, 247.5°, 292.5°, and 337.5° for discrimination accuracy) as within-subjects variables, and HPD type (Combat Arms^™^, Peltor^™^ H515FB, and double protection) as a between-subjects variable, on (1) discrimination accuracy (i.e., mean RMSE) and (2) mirror image reversal errors (i.e., percentage of localization to the opposite hemifield). Additional repeated measures ANOVAs and one-way ANOVAs were used when interactions were found significant. Post hoc analyses were performed using Least Significant Difference (LSD) tests. As HPDs significantly decrease localization accuracy, we chose not to compare performance with an HPD to the baseline (no-HPD), but to present the data separately, by comparing the relative baseline performance across different stimuli and, independently, comparing performance when utilizing various HPDs.

## 3. Results

### 3.1. Discrimination Accuracy

#### 3.1.1. No-HPD (Baseline)

**Figure 5:**
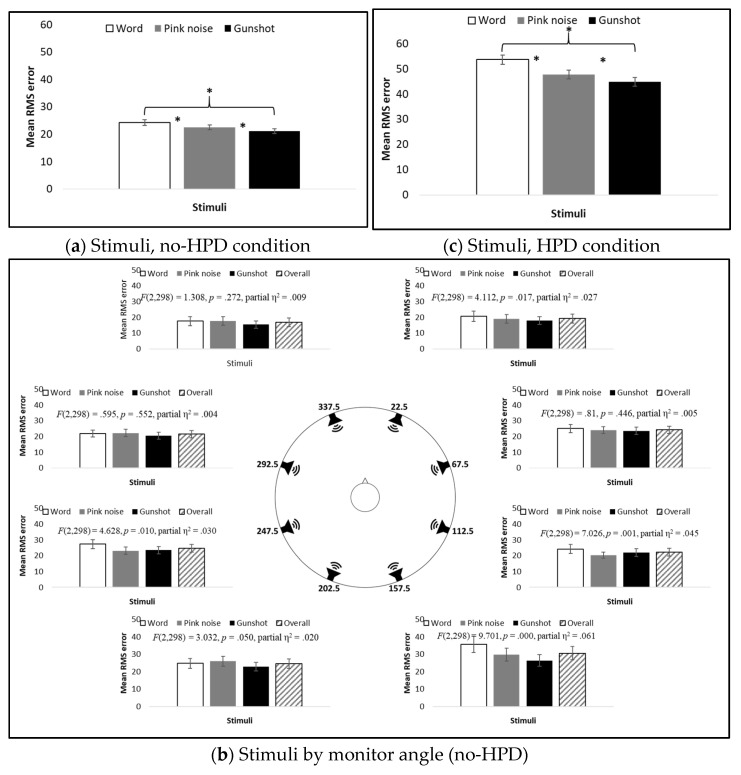

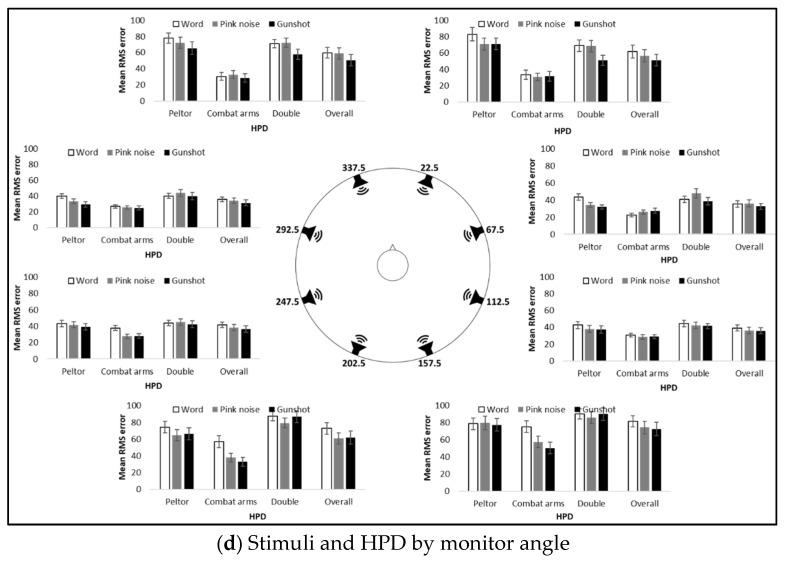
Mean RMSE of stimuli for (**a**) stimuli only (no-HPD condition), averaged across all 90 participants; (**b**) stimuli only (no-HPD condition) at each source angle, averaged across all 90 participants, including statistics for main effect for stimuli at each source angle; (**c**) stimuli only (HPD condition), averaged across HPDs for all 90 participants; and (**d**) stimuli and HPD at each source angle, averaged across 30 participants in each HPD group. * *p* < 0.05.

Main effects for RMSE were found for both stimulus type and source angle (*F*(2,172) = 13.304, *p* = 0.000, partial η^2^ = 0.134; and *F*(7,602) = 19.564, *p* = 0.000, partial η^2^ = 0.185, respectively). As expected, no difference in RMSE was found between HPD groups under the no-HPD condition (*F*(2,86) = 2.097, *p* =0.129, partial η^2^ = 0.047). The mean RMSE differed between all three stimuli, with the word having the largest RMSE, and the gunshot the smallest (Figure 5a). The results for source angles showed that, in general, stimuli originating from monitors located in the back (157.5° and 202.5°) had the largest RMSE, the front monitors (22.5° and 337.5°) had the smallest, and no difference in the mean RMSE was found between most of the stimuli originating from right (67.5° and 112.5°) and left (247.5° and 292.5°) monitors (Figure 5b, overall bar, and Table 1a). Interestingly, in almost all hemifields, there was a significant difference in RMSE between the two monitors positioned in the hemifield. For instance, in both the front and back hemifields, the right monitors (22.5° and 157.5°) had a larger RMSE than the left monitors did (337.5° and 202.5°), while in the left hemifield, the rear monitor (247.5°) had a larger RMSE than the front monitor did (292.5°). No difference in RMSE, however, was found between the monitors in the right hemifield (67.5°and 112.5°).

A significant Stimulus Type X Source Angle interaction was found (*F*(14,204) = 1.976, *p* = 0.017, partial η^2^ = 0.022). Separate repeated measures ANOVAs for each angle showed a significant main effect for stimulus type for only four of the eight source angles (Figure 5b). For all angles, the word had the largest RMSE and the gunshot had the smallest. Pink noise had a smaller RMSE than words did for source angles 112.5° and 247.5° and a larger RMSE than the gunshot did for source angle 157.5° (Table 2).

#### 3.1.2. With-HPD

Main effects were found for stimulus type, source angle, and HPD type (*F*(2,174) = 29.082, *p* = 0.000, partial η^2^ = 0.251; *F*(2,174) = 40.580, *p* = 0.000, partial η^2^ = 0.318; and *F*(2,87) = 37.222, *p* = 0.000, partial η^2^ = 0.461, respectively). As was observed in the no-HPD results, the RMSE differed between all three stimuli: the word had the largest RMSE and gunshot the smallest (Figure 5c). The mean RMSE for each stimulus at each source angle under the HPD condition, and the overall average across HPDs, are presented in Figure 5d. Post hoc LSD test results between source angles are presented in Table 1b. Similar to the results for the no-HPD condition, the largest RMSEs were found for monitors in the back (157.5° and 202.5°), although the back-left monitor (202.5°) had a smaller RMSE than the back-right one (157.5°) and a similar RMSE to the front monitors (22.5° and 337.5°). Under the HPD condition, the smallest mean RMSEs were observed in the right and left monitors (67.5°, 112.5°, 247.5°, and 292.5°). Combat Arms^™^ 4.1 resulted in the smallest overall mean RMSE (compared to Peltor^™^ H515FB: LSD = −21.009, *p* = 0.000; and compared to double protection: LSD = −24.422, *p* = 0.000). Importantly, no difference in the overall mean RMSE was found between Peltor^™^ H515FB and double protection (LSD = 3.413, *p* = 0.269).

Significant interactions were found for Stimulus Type X Source Angle, Source Angle X HPD Type, and Stimulus Type X Source Angle X HPD Type (*F*(14,1,218) = 2.010, *p* = 0.014, partial η^2^ = 0.023; *F*(14,609) = 3.587, *p* < 0.001, partial η^2^ = 0.076; and *F*(28,1,218) = 2.560, *p* = 0.000, partial η^2^ = 0.056, respectively). Separate repeated measures ANOVAs for each HPD at different source angles showed that the difference between stimuli is sensitive to HPD type and source angle (Table 3): for the two front monitors (22.5° and 337.5°), a main effect for stimulus type was found only in double protection, while for the two back monitors (157.5° and 202°), along with 247.5°, a main effect for stimulus type was found only for Combat Arms^™^ 4.1. Monitors at angles 67.5° and 292.5° displayed a main effect for stimulus type for Peltor^™^ H515FB. For all these effects, the word had a larger RMSE than the gunshot. In the front monitors, pink noise also had a larger RMSE error than the gunshot (Table 4).

### 3.2. Mirror Image Reversal Errors

#### 3.2.1. No-HPD (Baseline)

**Figure 6:**
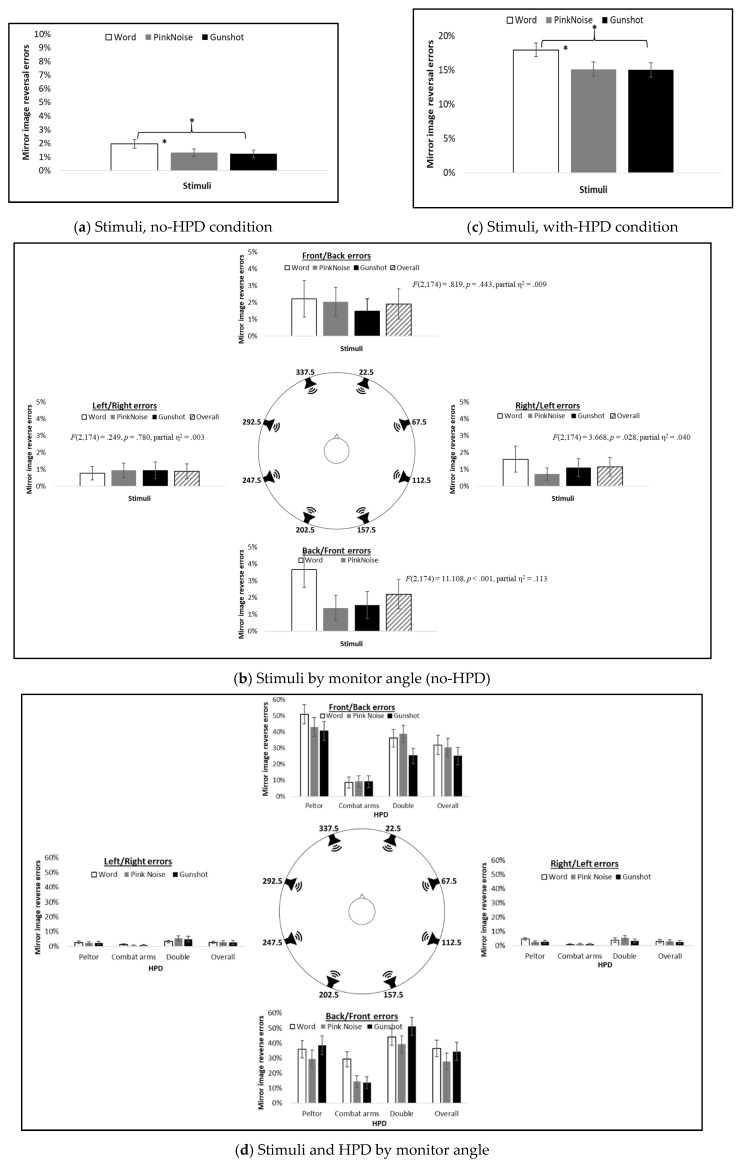
Mean percent of mirror image reverse errors for (**a**) stimuli only (no-HPD condition), averaged across all 90 participants; (**b**) stimuli only (no-HPD condition) at each hemifield, averaged across all 90 participants, including statistics for main effect for stimuli in each hemifield; (**c**) stimuli only (with HPD condition), averaged across HPDs, for all 90 participants; and (**d**) stimuli and HPD at each hemifield, averaged across 30 participants in each HPD group. * *p* < 0.05.

Main effects were found for stimulus type and hemifield, but not for HPD groups (*F*(2,174) = 7.442, *p* = 0.001, partial η^2^ = 0.079; *F*(3,174) = 5.823, *p* = 0.001, partial η^2^ = 0.063; and *F*(2,87) = 2.274, *p* = 0.109, partial η^2^ = 0.050). The word had the largest percentage of mirror image reversal errors (Figure 6a). In addition, there were more F/B and B/F errors than R/L and L/R errors (Figure 6b, overall bar, and Table 5a).

A significant Stimulus Type X Hemifield interaction was found (*F*(9,783) = 3.774, *p* = 0.001, partial η^2^ = 0.042). Figure 6b presents the mean percentage of mirror image reversal errors for different stimuli. Separate repeated measures ANOVAs for each hemifield showed significant main effects for stimulus type only for B/F and R/L errors, and not for F/B and L/R errors. In both B/F and R/L hemifields, the word had significantly more B/F and R/L errors than pink noise, and more B/F errors than the gunshot (Table 6a).

#### 3.2.2. With-HPD

Main effects were found for stimulus type, hemifield, and HPD type (*F*(2,174) = 11.589, *p* < 0.001, partial η^2^ = 0.118; *F*(3,174) = 58.829, *p* < 0.001, partial η^2^ = 0.403; and *F*(1,87) = 581.961, *p* < 0.001, partial η^2^ = 0.870, respectively). As with the no-HPD condition, the word had the largest percentage of mirror image reversal errors (Figure 6c). There were also more F/B and B/F reversal errors than R/L and L/F errors (Table 5b). The Combat Arms^™^ 4.1 had less errors than both the Peltor^™^ H515FB and double-protection HPDs did (LSD = −0.138, *p* < 0.001 and LSD = −0.142, *p* < 0.001, respectively).

Significant interactions were found for Stimuli Type X HPD Type, Hemifield X HPD Type, Stimuli Type X Hemifield, and Stimuli X Hemifield X HPD Type (*F*(4,174) = 3.703, *p* = 0.006, partial η^2^ = 0.078; *F*(6,261) = 5.965, *p* < 0.001, partial η^2^ = 0.121; *F*(6,522) = 6.909, *p* < 0.001, partial η^2^ = 0.074; and *F*(14,522) = 4.568, *p* < 0.001, partial η^2^ = 0.095). Figure 6d presents the mean percent of mirror image reversal errors by each HPD for the different stimulus types. Separate repeated measures ANOVAs showed main effects for stimuli perceived while using different HPDs only for F/B and B/F errors, with main effects for stimuli perceived with all HPDs for B/F errors, and only with Peltor^™^ and double protection for F/B errors (Table 6b). The word stimulus had the largest percentage of errors: more F/B errors than for the gunshot when using the Peltor^™^ or double-protection HPDs, and more B/F errors than both pink noise and gunshot when using the Combat Arms^™^ HPD. Pink noise displayed more F/B hemifield errors than the gunshot stimulus when using double-HPD protection, but less B/F errors than the gunshot when using the Peltor^™^ or double-protection HPDs (Table 6b).

## 4. Discussion

In the present study, we tested word localization, compared with pink noise and gunshot noise, considering the usage of various HPDs and the source angle/hemifield. Localization of a word was more difficult than the other stimuli. This finding was robust and consistent under conditions of HPD or no-HPD use, and when data were analyzed for the mean RMSE and for mirror image reversal errors. Evident as well was a difference in the effect of the sound source angle and hemifield on localization, depending on whether the listener used, or did not use, HPDs. With no HPDs used, localization was more accurate for sounds delivered from the front. However, with HPDs, the mean RMSE and mirror image reversal errors for stimuli delivered from the front increased, and localization was more accurate for stimuli delivered from the left or right. These findings demonstrate the sensitivity of localization to different characteristics of the environment, listener, and stimuli.

It is well-established that a stimulus’ spectrum, i.e., the energy content within the frequency bandwidth, affects the ability to localize it [3,17,19,23,30]. The stimuli used in this study—a word (/esh/), pink noise, and a gunshot—differed in their bandwidth and frequency region. Pink noise and the M16 gunshot (recorded at 200 feet (60.96 m) from the source) had wider bandwidths than the word did, and had higher energy levels in the low and high frequencies, as opposed to the word whose high energy level was mainly in middle-range frequencies. In addition, the accumulated energy of the gunshot and pink noise was much higher than that of the word, with the gunshot having 50% more accumulated energy than pink noise did, and the pink noise having about 50% more accumulated energy than the word did. Accordingly, the pink noise and gunshot were more accurately localized under both no-HPD and with-HPD conditions, with the gunshot being better localized than the pink noise.

The findings of the few previous studies that tested localization of speech stimuli among normal hearing participants support our findings. Jones et al. [11] tested both pink noise and monosyllabic consonant-nucleus-consonant words, similar to the word /esh/ that was used in the present study. However, their normal-hearing participants were tested in a *virtual* acoustic space, namely, stimuli were treated to simulate being delivered from points around the participant and were delivered through headphones. Studies show that localization in a virtual acoustic space is more difficult than in a real one [11,36]. Nevertheless, in line with our findings, they also showed that word localization was more difficult than pink noise, as evidenced by a smaller mean RMSE than for that of words. Other studies compared speech signals to white noise and showed that speech stimuli were localized more poorly than white noise [23,24,25]. Borg et al. [7] showed that gunshot noise was localized much better than a dog bark, which had a much narrower spectrum with almost no energy in high frequencies. Although a different speech content was tested, these studies, along with the present, show that speech stimuli are more difficult to localize than other stimuli.

A greater difficulty in localizing the word, in comparison with the pink noise and gunshot, was also evident when examining the sound source angle/hemifield. When the sound source was difficult to localize, such as from the back, or from the front when HPDs were used, the difficulty in localizing the word (relative to the pink noise and gunshot) was enhanced. These results show that, in spite of the interactions shown between stimulus type and source angle, and also with HPD use, the pattern of localizing different stimuli was maintained and changed only in its magnitude. Hence, these data indicate differential sensitivity to localizing varying stimuli at different angles. This point should be further studied, mainly in relation to the availability of different localization cues in different source locations.

In terms of localization, comparison between HPDs showed that, as expected, performance worsened with HPD use. The mean RMSE (across all stimuli, angles, hemifields, and HPDs) increased from 23° to 50°, and the mean mirror image reverse error increased from 2% to 16%, with HPD use. Nevertheless, the use of Combat Arms^™^ earplugs resulted in much smaller and fewer errors than those with Peltor^™^ earmuffs and double protection, especially for sounds originating from the front monitors. Indeed, while the mean RMSEs were smallest for the front monitors when HPDs were not used, when HPDs were used, the mean RMSEs for the front monitors exceeded the mean RMSEs for the right and left monitors. Previous studies also found better localization with earplugs than earmuffs [4,7,8,34,37].

Some studies attributed the difficulty in localization mainly to the attenuation level [8,26], while others attributed it to the limitation of pinnae cues [4,6]. The present study provides support to both lines of evidence. The general decline in localization accuracy when all types of HPDs were used is evidence for the effect of attenuation on localization. However, with HPD use, localization errors for sounds coming from the front and back increased more than sounds coming from the right or left. This serves as evidence for the effect of pinnae cues, or their limitation, on localization. In general, the localization of sounds from the right and left is achieved using interaural differences in time and level, while the localization of sounds from the front and back is achieved primarily using cues related to the pinnae, head, and torso. A larger increase in localization errors to sounds coming from the front and back, than from the right and left, implies a greater reduction in pinnae cues, rather than interaural cues.

In the present study, we analyzed the data in two ways that demonstrated participant ability, or, more accurately, inability—as both measures focused on errors—to localize various sounds under different conditions. The measure of mean RMSE is a classic way of analyzing localization and indicating precision level. The measure of mirror image reverse error demonstrates when the listener misses the appropriate localization cues. In real world scenarios, a mirror image reverse error reflects when a listener turns their head or body in the wrong direction; therefore, this measure can be considered more ecological than the mean RMSE. Brown [13] used a measure of Very Large Errors (VLEs), defined as errors larger than 45°; their purpose was also to present localization data ecologically by showing the conditions in which listeners will turn their head in the wrong direction. Other studies used the measure of mirror image reverse errors [7,8,9]. These measures can be very useful when predictions are made about listener behavior in real world situations.

Along these lines, the present study tested localization in a 360° array. Some previous researchers chose to study localization in only a partial array range, testing stimuli from the front or the side only [3,14,17,30,38,39]. This enables a focus on designated azimuths and selected cues. In the present study, however, we chose to study localization in a circular array to provide information on all cues and learn about listener behavior in a real-world situational design in which sound can be delivered from all directions around the listener. Thus, the current study’s data provide ecological predictions on localization.

The data provided by the current study have implications for both listeners who use HPDs, and those who do not. For both groups, it was difficult to localize the spoken word relative to the other stimuli. Localizing speech is especially critical in potentially dangerous situations, in any outdoor activity, at work, and in military training and combat, when erroneous localization of a single short word might be crucial. Speech localization is also critically important to aging adults whose hearing sensitivity deteriorates, or other hearing-impaired listeners. Moreover, for those who use HPDs, the importance of studying the localization of speech is demonstrated by the findings of Yehudai et al. [40] on the necessity of removing HPDs to improve communication in battle due to the difficulty of perceiving speech sounds (e.g., commands) while using HPDs. That said, for those who need to localize speech in loud environments, removing their HPDs can endanger their hearing. The current study’s findings suggest that when localization is important, earplugs should be preferred over earmuffs. A similar recommendation was previously suggested by Noble and Russell [4].

While the current study’s findings are in line with previous studies, they are applicable to the particular stimuli and HPDs that we tested. Therefore, exploration should be extended to other stimuli and HPDs. Aside from testing additional types of HPDs with different attenuation spectra and levels, testing different types of speech stimuli is also necessary, particularly words and sentences with different durations and spectra. In addition, following Derey et al. [37] who showed the effect of the sound category (vocalizations, traffic sounds, or tones) and behavioral relevance (neutral versus fearful sounds with differential effects on behavior) on localization, the effect of the content’s relevance and the speaker’s familiarity to the listener should also be tested. An additional limitation of the present study is that the attenuation level of HPDs was measured generally, using a free-field microphone, and not individually for each participant; adding the measurement of individual attenuation levels as a predicting factor for localization would be worthwhile.

In sum, the present study focused on the localization of three sounds and demonstrated more difficulty in localizing a monosyllabic word than pink noise and gunshot noise. In addition, the current study findings emphasize the importance of pinna cues in localization by demonstrating the effect of their reduction when using HPDs.

## Figures and Tables

**Table 1:**
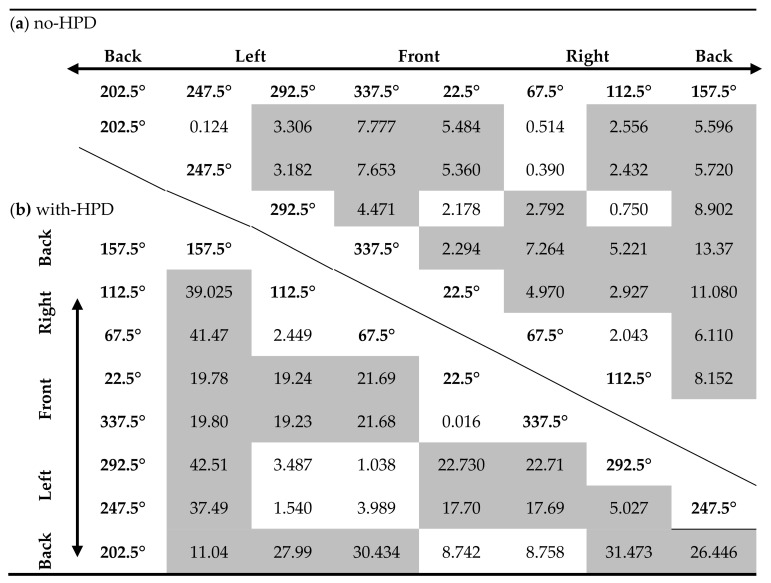
Results of Least-Significant-Difference (LSD) test for post hoc comparisons between the mean RMSE for monitors in different angles in the (a) non-HPD condition and (b) HPD condition (monitor angles correspond to Figure 1).

Significant comparisons are marked with shaded cells.

**Table 2:**
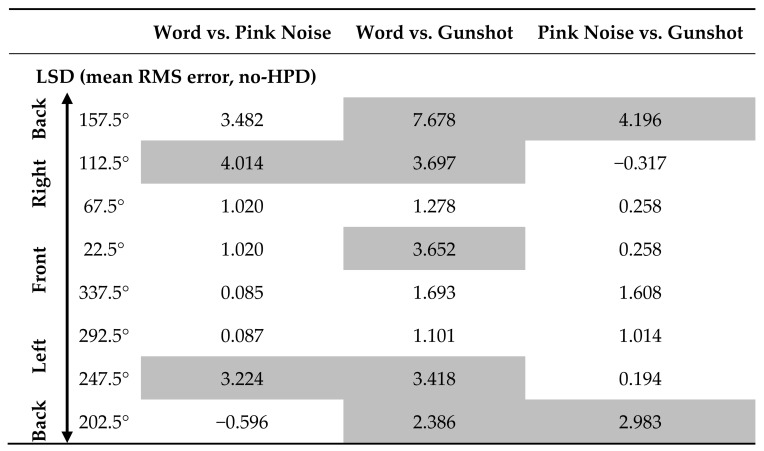
Post hoc LSD analyses between stimuli used in the study at each monitor angle.

Significant comparisons are marked with shaded cells.

**Table 3:**
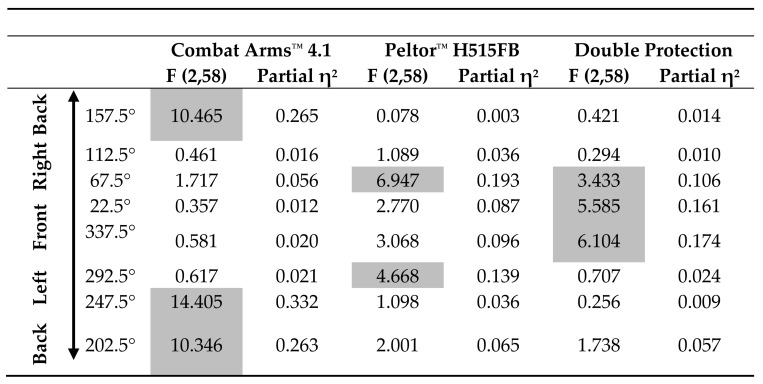
Main effects for comparison between stimuli using different HPDs and from different source angles.

**Table 4:** Post hoc analysis (LSD) for comparison between stimuli using different HPDs and from different source angles.

		Combat Arms^™^ 4.1			Peltor^™^ H515FB			Double Protection		
		Word vs. Pink Noise	Word vs. Gunshot	Pink Noise vs. Gunshot	Word vs. Pink Noise	Word vs. Gunshot	Pink Noise vs. Gunshot	Word vs. Pink Noise	Word vs. Gunshot	Pink Noise vs. Gunshot
**Back**	157.5°	17.744	24.854	7.110	−1.125	1.124	2.249	4.636	0.418	−4.219
**Right**	112.5°	2.108	1.834	−0.274	4.634	5.376	0.742	2.147	2.815	0.668
	67.5°	−3.403	−4.789	−1.386	9.263	11.597	2.334	−7.114	2.182	9.297
**Front**	22.5°	2.962	2.063	−0.899	12.268	12.115	−0.153	0.538	17.849	17.312
	337.5°	−2.300	1.817	4.117	5.659	12.485	6.826	−1.102	13.360	14.462
**Left**	292.5°	1.400	2.282	0.882	6.486	10.542	4.055	−3.730	−0.010	3.720
	247.5°	9.916	10.089	0.173	1.456	4.119	2.662	−1.316	1.340	2.656
**Back**	202.5°	18.819	23.774	4.955	9.445	7.831	−1.614	8.445	0.983	−7.462

*Note.* Significant comparisons are marked with shaded cells.

**Table 5:**
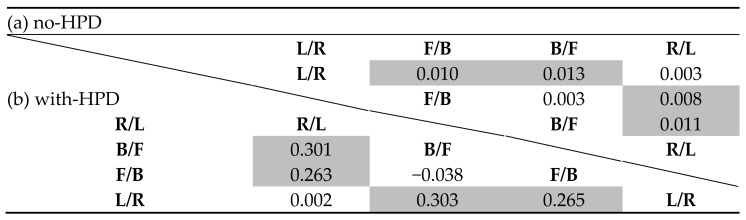
Post hoc LSD analyses between hemifields under (a) no-HPD and (b) HPD conditions.

*Note.* Significant comparisons are marked with shaded cells.

**Table 6:** Comparisons of mirror image reversal errors in each hemifield for the (a) no-HPD condition (LSD analyses between stimuli) and (b) HPD condition (main effects for each HPD, and post-hoc LSD analyses between stimuli for each HPD).

(a) no-HPD				
	**L/R**	**F/B**	**B/F**	**R/L**
Word vs. pink noise ^1^	−0.002	0.002	0.023	0.009
Word vs. gunshot ^1^	−0.002	0.007	0.021	0.005
Pink noise vs. gunshot ^1^	−0.001	0.006	−0.002	−0.004
				
(b) with-HPD				
	**L/R**	**F/B**	**B/F**	**R/L**
Peltor^™^^2^	0.027, 0.001	4.234, 0.127	3.830, 0.117	0.514, 0.017
Word vs. pink noise ^1^	0.001	0.080	0.065	0.008
Word vs. gunshot ^1^	0.001	0.103	−0.027	0.006
Pink noise vs. gunshot ^1^	0.007	0.023	−0.092	−0.002
Combat Arms^™^^2^	0.635, 0.021	0.099, 0.003	11.387, 0.282	0.125, 0.004
Word vs. pink noise ^1^	0.003	−0.007	0.148	0.000
Word vs. gunshot ^1^	0.002	−0.005	0.157	−0.002
Pink noise vs. gunshot ^1^	−0.002	0.002	0.008	−0.002
Double protection ^2^	0.329, 0.011	6.415, 0.181	4.258, 0.128	3.083, 0.096
Word vs. pink noise ^1^	−0.009	−0.027	0.047	−0.013
Word vs. gunshot ^1^	−0.003	0.110	−0.072	0.009
Pink noise vs. gunshot ^1^	0.007	0.137	−0.118	0.022

^1^ LSD test; ^2^ F(2,58), partial η^2^

*Note.* Significant comparisons are marked with shaded cells.

## Data Availability

The data presented in this study are available on request from the corresponding author. The data are not publicly available, due to ongoing analysis for the data.
